# Supplementary material for: Introduction of artificial plants has no detrimental or beneficial effects on laboratory zebrafish husbandry but limits available swimming space
Source: PLoS One. 2026 May 19;21(5):e0348591. doi: 10.1371/journal.pone.0348591 (PMC13186355; doi:10.1371/journal.pone.0348591)
Supplement: S1 Fig — (DOCX) [file pone.0348591.s003.docx]

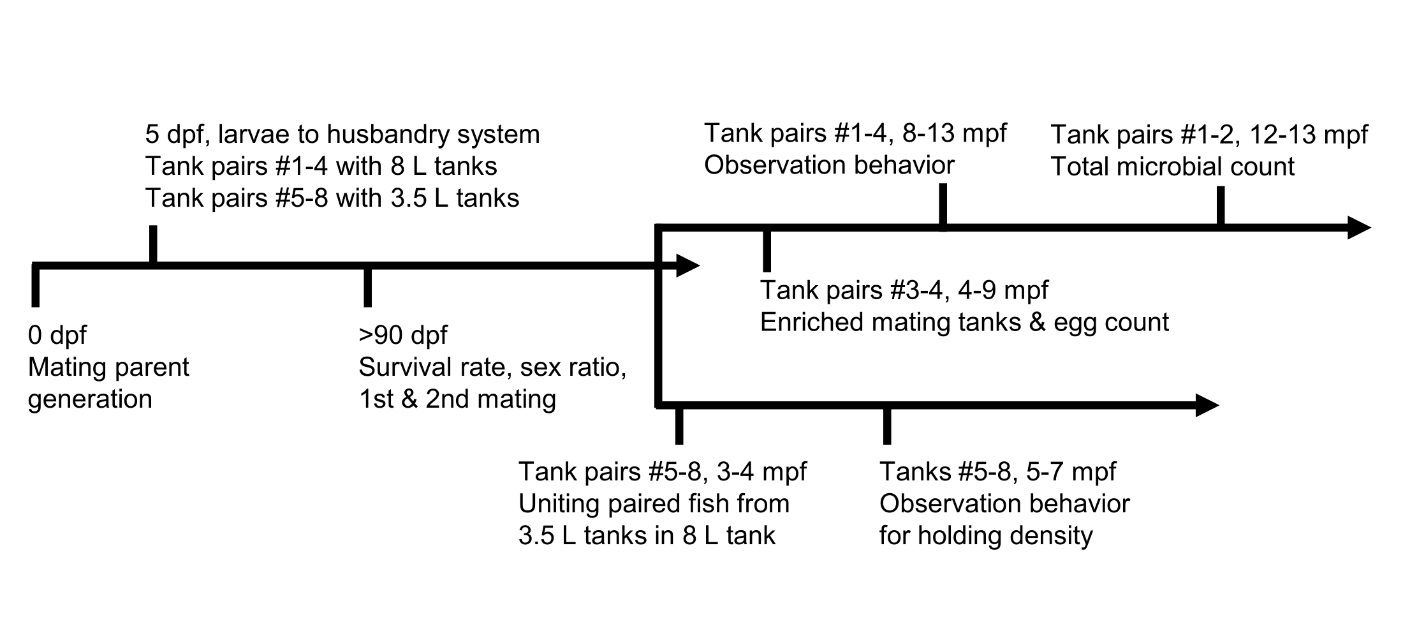


**S3 Fig: Timeline of events.** At 0 days post fertilization (dpf) new zebrafish lines were created by mating the parent generation. At 5 dpf larvae were transferred to the main system to paired tanks with either non-enriched or enriched conditions. Pairs #1-4 had 8 L tanks throughout. Pairs #5-8 had initially 3.5 L tanks. Around 90 dpf survival rate and sex ratio were determined and the first two mating were performed. Then fish from paired 3.5 L tanks #5-8 were joined in an 8 L tank, resulting in different holding densities. In the following months behavior in tank pairs #1-4 and tanks #5-8 was observed. In parallel tank pairs #3-4 were continuously used for mating with enriched mating tanks. Finally, water samples from tank pairs #1-2 were used to determine total microbial count at 12-13 months post fertilization (mpf).
