# Supplementary figures and images for: Introduction of artificial plants has no detrimental or beneficial effects on laboratory zebrafish husbandry but limits available swimming space
Source: PLoS One. 2026 May 19;21(5):e0348591. doi: 10.1371/journal.pone.0348591 (PMC13186355; doi:10.1371/journal.pone.0348591)

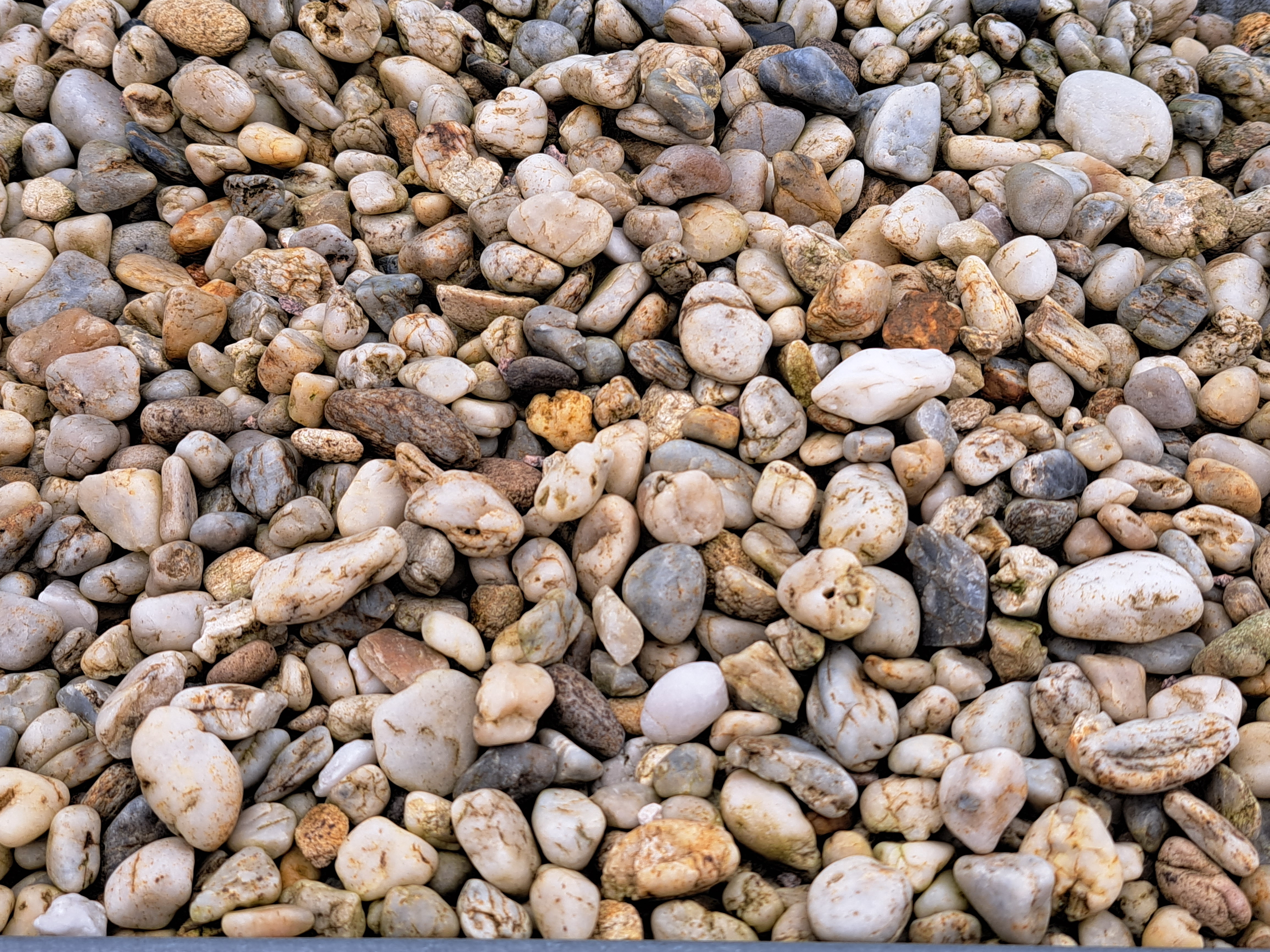

Supplement: S2 Fig — (JPG) [file pone.0348591.s004.jpg]
