# Supplementary material for: Introduction of artificial plants has no detrimental or beneficial effects on laboratory zebrafish husbandry but limits available swimming space
Source: PLoS One. 2026 May 19;21(5):e0348591. doi: 10.1371/journal.pone.0348591 (PMC13186355; doi:10.1371/journal.pone.0348591)
Supplement: Attachment — Submitted filename: Response to Reviewers.docx [file pone.0348591.s006.docx]

Dear Editor and Reviewers,

Thank you for reviewing our manuscript PONE-D-25-66088 and providing helpful comments and suggestions. We feel that it has now significantly improved and hope for your acceptance. Please see the detailed answers to the points raised below.

With best regards,

Nils Ohnesorge

**Editor remarks and Journal requirements**

*Both reviewers raise similar points, many of them concerning the interpretation and wording of your results. As one reviewer pointed out, the wording of the main headings are particularly important, since they are often read by people involved in regulatory offices with minimal scientific background.*

We agree. We have changed the main heading accordingly to: “Introduction of artificial plants had no detrimental or beneficial effects on laboratory zebrafish husbandry but limited available swimming space”

*If there are no restrictions, please upload the minimal anonymized data set necessary to replicate your study findings to a stable, public repository and provide us with the relevant URLs, DOIs, or accession numbers.*

We have deposited the raw data at the BioImage Archive under accession number S-BIAD2980, accessible under the following link: https://www.ebi.ac.uk/biostudies/bioimages/studies/S-BIAD2980?key=7dd22efe-beab-4a52-9a42-d6d51b5adc63

*We note that you have included the phrase “data not shown” in your manuscript. Unfortunately, this does not meet our data sharing requirements. […], if the data are not a core part of the research being presented in your study, we ask that you remove the phrase that refers to these data.*

We have removed the phrase that referred to these data.

**Reviewer #1 comments**

*Line 20/21 abstract: “It was shown that this can reduce stress and improve cognitive abilities”..this was a bit confusing as I expected that the paper itself generated this data, which was not the case…please change to “Other reports have…*

We agree. This was changed to: “Other reports have shown that this can reduce stress or anxiety and improve cognitive abilities, survival rate and fertility in these animals.”

*Line 27 avoid stating increases if not statistically significant, it should be: No statistically increase of survival was observed, also mention absence of significant benefit in terms of egg laying. Mention that the avoidance of plants was statistically significant.
I would weaken the final statement in the abstract in the light of the data presented by the paper itself.*

We have changed the wording of the paragraphs accordingly to better reflect the statistical evaluation of the experiments.

*Line 154: Plants that are present in the natural habitat of zebrafish: reference needed, this seems very difficult to verify, amend?*

We have added a reference regarding aquatic plants present in India and changed the sentence to the following: “Four of these aquatic plants were selected that mimic species present in India or Southeast Asia (23)…”

*Figure 1: Position of plant in 8 liter tanks should ideally also be shown in this figure (a top down view would be best), the rubber suction cup is not visible in C, but the main text suggests otherwise, please show detail, or a separate picture.*

We have added additional pictures to figure 1 to better illustrate the positions of plants and suction cup.

*Line 181 With less larvae available? Odd do you mean: If less than 25 larvae per tank were available…?*

Our apologies for this confusing phrasing. We changed the sentence to hopefully clarify as follows: “Either 25 larvae were raised in a 3.5 L tank or 30-37 larvae in an 8 L tank, with their numbers depending on overall needs for breeding these lines in the facility and mating success.”

*Line 186 Were the fish in the non-enriched tanks able to see the plants in the enriched tank? Could this visual stimulation have effects? It might have been better to visually isolate them. This might need some discussion.*

This is a valid point that we considered but eventually decided against to avoid larger changes in our husbandry setting in regards to handling the tanks, change of lighting or visual enrichment of siblings in neighboring tanks. In addition, the overall aim of the study was to evaluate effects of structural not visual enrichment but it certainly merits a follow up study. We added a paragraph discussing these limitations of our study in the Discussion section.

*Line 218 the picture regime in the quiet time is clear and makes sense however the regime during the day is unclear it sounds like only one picture was taken at 4pm, which will unlikely be sufficiently representativie of activity during the day..please make this more explicit*

We apologize for this confusion. For the daytime regime we evaluated in total 72 pictures of 8 different tanks (4 pairs) on 9 different days for both enriched and non-enriched tanks. That is 36 images for each enriched or non-enriched conditions. We changed the description and added the following for better clarity: “In total 144 picture were analyzed with 36 each for non-enriched or enriched tanks with or without human presence.”

*Line 223 “separate the tank equally either in left and right or in left, middle and right areas.” I understand from this that a 2D projection and not a 3D position was determined, this need to be made explicit, or clarified better, it also need to be clear where plants are in this setup. I realise there is a picture in Fig5..refer to this at this point in the text?*

We have clarified that a 2D projection was analyzed and added additional pictures to figure 1 to better illustrate the positions of the plants.

*Line 237 The precise raising and pooling strategies are hard to follow from the text, please add a arrow diagram/timeline in the supplementary data.*

We have added a timeline diagram as supplement figure 3.

*Line 284 Phrasing is misleading, here there is no significance. This needs to be changed to: The addition of plastic plants did not lead to a statistically significant increase or decrease in survival. Then provide calculations of which difference would be detectible with 90% security, using your spread in the data as a guide. (NB also the fact that enriched had better survival 6 out of 8 times is not statistically significant (>14%). In short, so give some indication of the sensitivity of your essay*

We agree. We have changed the sentence accordingly and added the following sentence: “Tanks enriched with artificial plants had no statistically significant improved survival rates of on average 77 %. Based on the standard deviation now known to be 11%, the effect size would have had to be 20 % to calculate statistical significance with 90 % certainty.”

*Line 288 The sex of the zebrafish is determined mostly by environmental factors: I do not think this is true, there are sex influencing loci as well, amend and/or provide references for such statements. (N.B. Here phrasing is neutral, as it should be.)*

It has been shown that holding density can have great influence on sex determination in laboratory zebrafish. We have changed the sentence accordingly and added an appropriate reference: “Holding density is a known factor to influence sex determination of the zebrafish during rearing (25), and structural enrichment might reduce the available swimming space and thereby the perceived holding density for the fish.”

*Discussion and overall conclusions:*

*In the final discussion it is made clear that there are no significant effects from the plants, apart from the fact that the fish are shown to avoid the plants. As an argument in favour of using them despite this, other papers are used (eg ref 14), I disagree with this way of drawing conclusions as it based on data that are gathered often under very different conditions, often testing fish in isolation, which is not what happens in stock maintenance.*

*The discussion and abstract advocate measures that are not supported by the author’s own measurements. Even though the required effort may be considered small, introducing plastic plants across thousands of tanks in 100s of facilities should only be supported if advantages are directly observed, with clear statistical significance.*

We tried to use the most appropriate references reflecting the situation in husbandries best whenever possible. We agree that one has to be careful to draw conclusions from experiments with single fish or small groups but in the absence of more appropriate data these finding should not be ignored either. Therefore, our main conclusion remains that more facilities need to test on effects of structural enrichment to fill this data gap and then decide based on their own findings if continued use is appropriate for them. We apologize, if we were misunderstood in advocating for an immediate and permanent use of structural enrichment for other facilities. We changed abstract and discussion accordingly to hopefully clarify on this distinction.

*Even more importantly, only thing that does show a significant result, is avoidance of the plants, to me this indicates that the fish do not like them and it causes artificial crowding. Place preference has been used extensively, as a way to measure aversion in behavioural studies, including several studies in mentioned in ref 14. I take from the data that lab-bred zebrafish maybe are adapted and are (mis)perceiving plants as a threat rather than an enrichment. This is surprising and interesting, and it would be extremely interesting to know if newly wild-caught zebrafish show the same behaviour, it would be very interesting to collaborate with labs (eg in india) who would have acces to such fish. This could be part of a discussion*

We included two references from Spence et al. (12) and Goshal et al. (35) who saw no difference in the use of structural enrichment between laboratory and wild-caught zebrafish. But we agree that data of zebrafish’s place preference and their use of structures in their environment in their natural habitat would be very valuable indeed, if this could be investigated. We have added comments to the Discussion section accordingly.

*Plants hinder visual health checks; the discussion should weigh the (un)measurable positive enrichment effect against the negative effect of (even if this happens rarely) missing a sick fish in visual inspections that may then perish and experience severe suffering*

We agree. We have expanded on the discussion regarding health benefits or risks posed by structural enrichment accordingly.

*Overall, I conclude that this study is certainly worthy of publishing however the tone of the article needs reflect the data that the paper obtains, and I think it should have the following title, which fits the data that were produced by the paper:*

*“Introduction of artificial plants does not give significant benefit during raising and breeding, but causes an aversive response in laboratory strains of zebrafish housed under standard conditions”*

*It is very important that the headline of this paper is right, because it may be read by people who make high-impact policy decisions, but do not go beyond title/abstract level.*

We thank you for this assessment and hope that by following your helpful suggestions we could change the manuscript to a more appropriate tone. We have decided for a more concise main title to address your comment as follows: “Introduction of artificial plants had no detrimental or beneficial effects on laboratory zebrafish husbandry but limited available swimming space”.

**Reviewer #2 comments**

*1. The Introduction is quite long and repeats several general arguments about enrichment, results from previous studies and lack of data. Consider revising to improve clarity and impact.*

We have shortened several paragraphs in the introduction to have a clearer and more concise text.

*2. This study is based on the usage of different genetic lines introduced in Methods. However, in the results these are named “line 1”, “line 2”, etc. without specifying what they are or without interpreting results in the light of these different genotypes. If these groups are genetically distinct, then we would expect some reference to what they are alternatively the term “cohort” or “group” would be a more appropriate to describe them.*

*For exapample in the discussion: “The survival rate was especially low in the last case observed (line 6). This was likely due to a bad quality of this clutch as the parent line was already old and had several unsuccessful matings before a small number of eggs was obtained. These eggs were below standard quality and had to be raised nonetheless to maintain the line.”…What is line 6? Does this correspond to a specific genotype? What is the age of these fish? Can line 6 be compared with another line of the same age that showed a better outcome?*

We have given background information like genetic status of the animals in the method section as well as in more detail in Supplement Table 2. We felt that our sample numbers were too small to investigate for line differences and instead opted for a pairwise comparison of sibling tanks, were line-specific difference should be mostly eliminated when averaged with other lines. Still, we included the information to be of use for future studies.

*3. In addition, the manuscript alternates between “lines,” “tanks,” and “conditions,” which make the experimental design difficult to follow. Using consistent and clearly defined terminology would greatly improve clarity.*

We agree and apologize for this unnecessary confusion. We considered the tanks as experimental unit and changed all mentions of “lines” and “conditions” in this context to “tanks” throughout the manuscript for better clarity.

*4. The authors should indicate in the text the total number of fish per “line/tank” and/or condition. As it stands, the frequent reference to ‘lines 1–6’ without clear reference to actual animal numbers makes it difficult to appreciate sample size and study scale
For example “Eight tanks of lines 1 and 2”, What does this indicate? four each, 2/6 or 8 each? Also” Over the course of one year six different lines have been raised in 16 tanks in the same husbandry system. Of every line half the tanks received structural enrichment in the form of artificial plants while the other half were kept under our standard conditions.” Here we have no idea of tank distribution per line and how many fish…."*

In tanks that were paired there were always similar number of fishes and by pair-wise comparison effects due to animal numbers should be mostly eliminated. Therefore, we have left out this information for most part of the manuscript and placed it in the supplements. We have included this information on animal numbers now for our behavioral observations, where this could be of greater interest to the reader.

*5. Statements such as ‘in 6 of 8 cases the survival rate was better with structural enrichment’ read somewhat anecdotal, especially in the absence of statistical significance. It would be preferable to focus on effect sizes, and to avoid more colloquial formulations that may overstate the value of a trend that is statistically non‑significant.*

We agree and have removed such mentions and instead focused more on statistical significance.

*6. The comparison between “standard” and “plant-enriched” environments is potentially confusing, as “standard” is vague and context-dependent, and may imply an optimal condition rather than a descriptive one. A clearer formulation would be: “Fish were raised under either non-enriched housing conditions (no structural enrichment) or enriched housing conditions, which included artificial plants.” This wording would avoid ambiguity.*

We have initially avoided “non-enriched” as label as by our usual husbandry settings these fish got several forms of enrichment as well, like gravel pictures under the tank, artemia as live food and visual enrichment of other fish in neighboring tanks. But we agree to your reasoning that “standard” could be potentially misunderstood as well and therefore changed the label “standard” to “non-enriched” throughout the manuscript.

*7. Importantly, this study distinguishes several genetic lines, each split into tanks with and without plants, which is sensible biologically. However, this fragments the data and further reduces power. Given that the main question is whether plant enrichment affects outcomes at all, it would be informative to analyse all tanks together as ‘enriched’ vs ‘non‑enriched’, while including line as a factor (or random effect) rather than running effectively separate mini‑studies per line (without however taking genotypes into account for interpretation). The current presentation, where ‘lines’ and ‘tanks’ are repeatedly described but rarely ummarized into a single enrichment effect across lines, makes it harder for the reader to see the overall impact of enrichment on the fish. Re‑analysing the data with all tanks grouped as ‘enriched’ vs ‘non‑enriched’, and treating line as a factor, is unlikely to change the general conclusion (no strong effect), but it would provide a clearer and statistically more robust estimate of the overall enrichment effect than the current fragmented, line‑by‑line presentation. Have the authors consider this? Can they comment on this?*

We think that this is a misunderstanding how we statistically analyzed our data. We only showed individual data points, like the bars of survival rates in figure 3A for greater transparency. Significant changes between individual non-enriched and enriched tanks were not calculated at this point. Instead, e.g. the mean survival rate and the difference between paired tanks were calculated and only then a t-test was applied to see if there was a significant change (Fig 3B). Therefore, there has been an overall analysis of all tanks combined.

In addition, the statistical approach of using paired tanks likely has increased statistical power. As it can be appreciated in Figure 3A, results from paired tanks are often quite similar but differ strongly between different pairs and our statistical tests confirmed that these pairing was indeed highly significant. This means that enrichment had overall less effect on the survival compared to other factors like genetics or the environment during the timepoint of raising the larvae. By subtracting the survival rate of a non-enriched tank with the rate of its paired enriched tank, theoretically all other confounding factors are removed except the enrichment itself, resulting in more power.

We have added descriptions in the method and result sections to clarify our approach using paired-tanks.

*8. Linked to the previous point, I am not an expert here, but to my understanding survival, sex ratio, and mating success are binomial (yer/no), and the use of t-test–based power calculations is not optimal for such bounded data, especially with small numbers of tanks; statistical methods designed for proportions would be more appropriate.*

It is true that discrete data points like number of individual females would require different forms of statistical analysis. In our case, we have compared the averages of different groups, like the ratio of females per tank which constitutes a form of continuous data that allows for the use of t-tests.

*9.Behavioral conclusions about welfare in particular stress are largely indirect because no direct measures were collected. Considere tuning down*

We agree. We have changed the wording of our conclusions to a more careful tone.
